# Associations between psychotic experience dimensions and polygenic liability to schizophrenia in a longitudinal birth cohort

**DOI:** 10.1192/bjo.2025.10825

**Published:** 2025-09-08

**Authors:** Alastair G. Cardno, Hein Heuvelman, Sophie E. Legge, James T. R. Walters, Stanley Zammit, Hannah J. Jones

**Affiliations:** Division of Psychological and Social Medicine, University of Leeds, UK; Department of Public Health, Policy and Systems, Institute of Population Health, University of Liverpool, UK; Centre for Neuropsychiatric Genetics and Genomics, Cardiff University, UK; Medical Research Council Integrative Epidemiology Unit at the University of Bristol, Population Health Sciences, Bristol Medical School, Bristol, UK; NIHR Bristol Biomedical Research Centre, University Hospitals Bristol and Weston NHS Foundation Trust, Bristol, UK; Centre for Academic Mental Health, Population Health Sciences, Bristol Medical School, University of Bristol, UK

**Keywords:** ALSPAC, CAPE, psychosis, schizophrenia, genetics

## Abstract

**Background:**

Some psychotic experiences in the general population show associations with higher schizophrenia and other mental health-related polygenic risk scores (PRSs), but studies have not usually included interviewer-rated positive, negative and disorganised dimensions, which show distinct associations in clinical samples.

**Aims:**

To investigate associations of these psychotic experience dimensions primarily with schizophrenia PRS and, secondarily, with other relevant PRSs.

**Method:**

Avon Longitudinal Study of Parents and Children (ALSPAC) birth cohort participants were assessed for positive, negative and disorganised psychotic experience dimensions from interviews, and for self-rated negative symptoms, at 24 years of age. Regression models were used to investigate associations between psychotic experience dimensions and schizophrenia and other PRSs (2500+ participants for each analysis).

**Results:**

Against expectation, none of the positive, negative or disorganised dimensions was associated with schizophrenia PRS. In secondary analysis, self-rated negative symptoms were associated with higher depression (*β* = 0.10 [95% CI 0.06–0.15]), anxiety (*β* = 0.09 [95% CI 0.04–0.13]), neuroticism (*β* = 0.11 [95% CI 0.06–0.15]) and autism (*β* = 0.09 [95% CI 0.05–0.13]) PRSs (all *P* < 0.001); and first-rank delusions were nominally associated with higher schizophrenia PRS (odds ratio 7.35 [95% CI 2.10–25.77], *P* = 0.002), although these experiences/symptoms were rare.

**Conclusions:**

Positive, negative and disorganised psychotic experiences are probably not strongly associated with polygenic liability to schizophrenia in this general population cohort of young adults. Self-rated negative symptoms may indicate social withdrawal/low motivation due to higher polygenic liability to affective disorders or autism, and first-rank delusions may indicate higher polygenic liability to schizophrenia, but these findings require independent confirmation.

Schizophrenia and related psychoses show considerable symptom variation, whose pattern of co-occurrence can be summarised by factor analysis into three main dimensions of broadly defined psychotic symptoms: positive (delusions and hallucinations), negative (restricted speech and affect, reduced motivation and socialisation) and disorganised (formal thought disorder, incongruous/inappropriate affect and bizarre behaviour).^1^ Subdivisions or merging of dimensions can also occur, depending on which symptoms are included,^2–4^ e.g. first-rank symptoms as a subdivision of the positive dimension, or a combined negative/disorganised dimension. Affective symptoms^5^ and cognitive abilities^6^ are also sometimes included in analyses.

There are notable differences in symptom dimension associations, which may reflect some aetiological differences. For example, positive symptoms are most responsive to antipsychotic medication,^7^ negative symptoms are most strongly associated with chronic illness course^8^ and disorganised symptoms show the most consistent evidence for substantial heritability.^9,10^ This highlights the value of investigating positive, negative and disorganised dimensions as separate, although related, phenotypes.

In addition to clinical symptoms, a range of milder psychotic experiences occur. In view of this, research based on clinical samples can be complemented by investigations of longitudinal birth cohorts,^11,12^ which include (a) a relatively well-defined population; (b) prospective assessments of risk factors and a broad range of relevant phenotypes from subclinical psychotic experiences to symptoms; and (c) the opportunity to observe how associations develop with age and less influence from factors such as medication effects, that can occur in clinical samples.

Longitudinal population-based research is being conducted, but often with questionnaire measures of experiences/symptoms or in relatively small samples, whereas assessments in clinical samples are normally based on responses to structured or semi-structured interview questions, interviewers’ observations and review of clinicians’ observations in case records.

The Avon Longitudinal Study of Parents and Children (ALSPAC) birth cohort^12–14^ (http://www.bristol.ac.uk/alspac) is distinctive, in that it includes large-scale data on psychotic experiences/symptoms from both semi-structured interview questions and interviewers’ observations up to age 24 years, around the peak age at onset of schizophrenia.^7^ The data including interviewers’ observations have not been previously studied, and are valuable for assessing negative and disorganised symptoms. This makes ALSPAC well suited for investigation of psychotic experience/symptom dimensions and their associations with schizophrenia risk factors, the main focus being on genetic risk factors in the current study.

## Genetic risk factors

Schizophrenia has a high heritability, of 60–80%.^7,15^ Among psychotic symptom dimensions, the disorganised dimension shows most consistent evidence of significant familial aggregation^2,9^ and has substantial twin heritability (∼80%),^10^ while the positive and negative dimensions show less consistent familial aggregation.^9^

In molecular genetic studies, higher scores on disorganised, negative/disorganised and sometimes negative dimensions are associated with a higher schizophrenia polygenic risk score (PRS),^4,6,16,17^ a measure of genetic loading for schizophrenia due to common genetic variants.

Additionally, the positive dimension is associated with schizophrenia PRS in some,^5,18–20^ but not all,^21,22^ studies that include other psychotic or bipolar disorders.

Psychotic experiences/symptoms in the general population show factor-derived dimensions similar to clinical samples,^11,14^ (although observer-rated disorganised symptoms have usually not been included) and are associated with family history of psychosis.^11^ Schizophrenia PRS has been associated with the positive and negative dimensions^13,14,23–25^ and self-rated cognitive disorganisation.^23^ Most studies have been based on self- or parent-rated questionnaires. One study based on a schizotypy interview found associations with positive and negative/disorganised dimensions.^26^ A further study investigated positive, negative and disorganised dimensions using a clinical research interview but found no significant associations, possibly because of the small sample size (*n* = 148).^27^

Previous investigations in ALSPAC have shown an association between schizophrenia PRS and the negative dimension at age 16 years, as assessed by the self-rated Community Assessment of Psychic Experiences (CAPE) questionnaire^28^ and defined as a categorical cut-off score,^13^ and by quantitative factor scores from correlated factors and bifactor models.^14^

Schizophrenia PRS was also associated with the positive dimension in the correlated factors model,^14^ based on self-report at 16 years.

Additionally, in a study that combined ALSPAC and other samples, schizophrenia PRS was associated with a parent-rated negative dimension at 16 years.^23^

In summary, in schizophrenia samples polygenic loading for schizophrenia is most associated with the disorganised or negative/disorganised dimensions. In samples including other psychoses and population-based samples there is sometimes an additional association with the positive dimension, possibly because these samples include a broader range of positive experience/symptom severity.

However, associations between psychotic experience dimensions and schizophrenia PRS may not be specific, because they have also been found with other PRSs, including those for depression, neuroticism and autism.^14,23–25^

In the current study, we extended the ALSPAC investigations to include four primary dimensions derived from (a) interview-based positive psychotic experiences (defined broadly from mild experiences to symptoms), (b) interviewer-observed negative symptoms, (c) interviewer-observed disorganised symptoms and (d) self-rated negative symptoms; to assessments up to 24 years; and to a newer, more powerful, schizophrenia PRS.^29^

## Study aims

The primary aim of this study was to investigate associations between psychotic experience dimensions assessed at 24 years and schizophrenia PRS. We hypothesised that all three dimensions based on interview would be associated with schizophrenia PRS, with effect sizes disorganised > negative > positive. We additionally hypothesised that the self-rated negative dimension, based on the CAPE questionnaire, would be associated with schizophrenia PRS at 24 years, as previously found in ALSPAC at 16 years.^13,14^

Secondary aims were to: (a) investigate associations of individual psychotic experiences with schizophrenia PRS, in case any were hidden by being combined in broader experience dimensions; (b) investigate associations of psychotic experience dimensions with other relevant PRSs, to assess how specific any associations with schizophrenia PRS were; (c) investigate the broader profile of associations of psychotic experience dimensions with demographic variables, developmental risk factors and affective disorders, in order to further contextualise the dimensions and their PRS associations; and (d) investigate the profile of these associations with psychotic disorder, in order to highlight any notable differences between psychotic experiences and symptoms.

## Method

### Study sample

Pregnant women residing in Avon, UK, with expected dates of delivery between 1 April 1991 and 31 December 1992 (enrolled, *n* = 14 541; live births alive at 1 year, *n* = 13 988), were invited to take part in ALSPAC.^30–32^ An additional 913 children who would have been eligible but whose mothers did not enrol during pregnancy were enrolled after age 7 years, resulting in a total sample of 14 901 children alive at age 1 year. There were 3866 participants at 24 years (56% female, 97% of White ethnicity).^12^ Between age 12 and 24 years, 313 (8.1%) had had a definite positive psychotic experience and 109 (2.8%) had a psychotic disorder.^12^

Study data were collected and managed using Research Electronic Data Capture (REDCap) electronic data capture tools hosted at the University of Bristol.^33^ REDCap is a secure, web-based software platform designed to support data capture for research studies.

The study website contains details of all the available data through a fully searchable data dictionary and variable search tool (http://www.bristol.ac.uk/alspac/researchers/our-data/).

The authors assert that all procedures contributing to this work comply with the ethical standards of the relevant national and institutional committees on human experimentation, and with the Helsinki Declaration of 1975 as revised in 2013. All procedures involving human subjects/patients had ethical approval from the ALSPAC Ethics and Law Committee and the Local Research Ethics Committees (details at http://www.bristol.ac.uk/alspac/researchers/research-ethics/; approval for 24-year assessments: National Research Ethics Service Committee South West – Frenchay: no. 14/SW/1173). All participants gave written informed consent.

### Assessment of psychotic experience/symptom dimensions

Three psychotic experience/symptom dimensions were derived from interview and one from questionnaire.

Interview-based psychotic experience/symptom dimensions were defined in terms of the semi-structured Psychosis-Like Symptom Interview (PLIKS)^12^ questions and interviewer observations at 24 years. Interview questions asked about experiences and symptoms occurring between 12 and 24 years (see Supplementary Method available at https://doi.org/10.1192/bjo.2025.10825 for further details regarding PLIKS).

We defined psychotic experience dimensions based on the most consistent core psychotic symptoms in dimensions derived from factor analysis of clinical samples^1,6^ and psychotic experiences in population samples.^11,14^ Psychotic experiences were defined to match as closely as possible core symptoms from our studies of clinical psychotic symptom dimensions,^8^ in order to facilitate comparison between the current population-based study and studies of clinical samples. In these studies, each dimension comprised two core symptoms (e.g. hallucinations and delusions for the positive dimension) and was scored from 0 to 2 according to how many of the core symptoms were present.

The overarching term psychotic experiences was used broadly in this study, to include both milder subclinical experiences and also symptoms (where there was significant distress or impairment), both subjective experiences described at interview, or in a questionnaire, and behaviour observed by interviewers. Within this, we referred to positive psychotic experiences, negative symptoms and disorganised symptoms, to follow common usage.^11,13,14^

Definitions of psychotic experience dimensions were as follows:

- positive dimension (Pos, scored 0–2): comprising any auditory or visual hallucination experience (score 1) and any delusion experience, including thought insertion/withdrawal/broadcast (score 1), rated at interview as definitely present and occurring between 12 and 24 years. Experiences that occurred only when waking up/falling asleep, with fever or under the influence of alcohol/drugs were not included;
- negative dimension (Neg, scored 0–2): comprising reduced interaction/speech (score 1) and restricted affect (score 1), rated as clearly present at interview;
- disorganised dimension (Dis, scored 0–2): comprising incoherent speech (score 1) and odd/inappropriate behaviour (score 1), rated as clearly present at interview;

See Supplementary file ‘Observed ratings from interview’ for the Neg and Dis symptom rating guide.

Neg and Dis symptoms were more likely to be observed if they were frequently occurring or easily evoked by the effort of being interviewed than if they were occasional and transient.

The distributions of dimension scores are shown in Table 1. Because the number of participants with scores of 2 was low, we dichotomised scores into a broad definition (regarded as present if either of the core experiences was present: score 0 versus 1–2). This was the primary definition for analysis.

**Table 1:** Distribution of psychotic experience dimension scores from interview assessments

Psychotic experience dimension score^a^	Psychotic experience dimension, *n* (%)		
	Positive^b^	Negative^c^	Disorganised^d^
0	3652 (94.6)	3695 (96.6)	3765 (98.4)
1	177 (4.6)	75 (2.0)	51 (1.3)
2	33 (0.9)	55 (1.4)	10 (0.3)
Total	3862	3825	3826

a. Psychotic experience dimension score based on the number of experiences/symptoms assessed as being present.

b. Positive dimension comprised hallucination and delusion experiences assessed by interviewer as being present at 12–24 years.

c. Negative dimension comprised reduced interaction/speech and restricted affect observed at interview at 24 years.

d. Disorganised dimension comprised incoherent speech and odd/inappropriate behaviour observed at interview at 24 years.

We also employed a narrow definition (regarded as present if both core experiences were present: score 0–1 versus 2). If there were substantial numbers of participants scoring 1 and 2, we would expect variables associated with a dimension to show an association with the broad definition and an association of a similar or greater effect size with the narrow definition, as seen in clinical samples.^8^ However, in the current study we confined investigation of the narrow definition to secondary analysis due to the low number of participants scoring 2 for dimensions, and in case the results could be combined with those from other samples in future meta-analyses.

We have dichotomised in a similar way to this in previous research on clinical samples where symptom dimension scores of 2 were uncommon.^8^

Additionally, broad Pos is similar to the presence/absence of any psychotic experience as used in other ALSPAC investigations,^13,34^ allowing contextualisation and comparison with results of these studies.

1. Self-rated negative dimension (self-rated Neg, scored 0–30): comprising the sum score of the 10 items relating to low emotional expression, motivation and socialisation in the CAPE questionnaire^28^ (each scored 0–3: never, sometimes, often, nearly always, respectively), completed at 24 years, regarding symptoms in the past month.

See Supplementary Method for further details about the CAPE questionnaire and Supplementary file ‘CAPE questions’ for the questions used.

We defined dimensions as simple sum scores, rather than conduct a factor analysis and derive factor scores, because of the different time frames and modes of assessment for the various experiences. Also, compared with factor scores, sum scores were easier to interpret and compare with the results of studies in clinical samples where corresponding symptom definitions were used.

Spearman correlations between dimensions ranged from 0.02 (Neg and self-rated Neg) to 0.36 (Neg and Dis; Supplementary Table 1).

### Polygenic risk score (PRS) analysis

The primary analysis was of schizophrenia PRS, derived from the White European ancestry sample of the PGC3 schizophrenia Genome-Wide Association Study (GWAS).^29^

PRSs were calculated for each ALSPAC individual using PLINK version 1.07 (https://zzz.bwh.harvard.edu/plink)^35^ by summing the number of risk alleles for each single nucleotide polymorphism (SNP) weighted by GWAS discovery sample effect size.

See Supplementary Method for further details on the genetic data and PRS calculation.

We used standardised scores generated from a list of SNPs, with a GWAS discovery sample *P*-value threshold (*P*_T_) ≤ 0.05 in regression models, to evaluate associations between PRS and psychotic experience dimensions: logistic regression for the interview-based dimensions, and linear regression for self-rated Neg, with CAPE scores square-root transformed due to the positive skew of data distribution. The transformation optimised the phenotype for linear regression analysis, but with the trade-off of reducing variability, especially of higher scores, and making real-world interpretation of the results less straightforward.

We restricted the PRS analysis to participants of White European ancestry (97% of the sample) and adjusted for gender and the first 10 population genetic ancestry principal components.

We treated primary analysis associations as statistically significant at *P* < 0.05, two-tailed, following adjustment for a Benjamini–Hochberg false discovery rate (FDR; 0.05) based on analyses of the four primary psychotic experience dimension phenotypes, in SPSS version 29 for Mac (IBM, Armonk, NY, USA; https://www.ibm.com/spss).

### Secondary analysis

In case any associations with individual psychotic experiences were hidden within the dimension results, we conducted associations between schizophrenia PRS and the presence/absence of the six individual psychotic experiences from the PLIKS interview that contributed to the dimensions, using logistic regression as in the primary analysis.

In order to assess how specific the schizophrenia PRS associations were, we conducted secondary analysis of associations between psychotic experience dimensions and other potentially relevant PRSs available in ALSPAC at the time of analysis: psychotic experiences in middle-aged/older adults, depression, anxiety, neuroticism and autism. We used the same methods as for the schizophrenia PRS analysis.

To give context and possible insights into the primary results, we also investigated associations of the psychotic experience dimensions with demographics, developmental risk factors, affective disorders, quality of life and social rapport variables. Finally, we conducted the primary and secondary analyses using psychotic disorder as the phenotype to gain insights into similarities and differences compared with broader positive psychotic experiences. See Supplementary Method for further details.

Analyses were conducted using logistic or linear regression models, with the psychotic experience dimension or psychotic disorder as the dependent variable and the demographic/risk factor as the independent variable, adjusted for gender (except the analysis of gender), in SPSS version 29 for Mac.

For all secondary analyses, in the results tables we indicate those results that are statistically significant at *P* < 0.05, *P* < 0.01 and *P* < 0.001, two-tailed and, in the text description and discussion of these results, focused on those with *P* < 0.001, describing these as associated while describing results at *P* < 0.01 as nominally associated.

## Results

### Associations between psychotic experience dimensions and schizophrenia PRS

The results of the primary analysis of associations between the four psychotic experience dimensions and schizophrenia PRS are shown in Table 2, with further details and descriptive statistics in Supplementary Tables 2–6 and Supplementary Figs 1 and 2. None of the associations was statistically significant.

**Table 2:** Regression analysis of psychotic experience dimensions on polygenic risk scores^a^

Polygenic risk score (PRS)	Interviewer-rated positive psychotic experiences^b^		Interviewer-rated negative symptoms^c^		Interviewer-rated disorganised symptoms^d^		Self-rated negative symptoms^e^	
	*n*	OR (95% CI)	*n*	OR (95% CI)	*n*	OR (95% CI)	*n*	*β* (95% CI)
Primary analysis								
Schizophrenia	2521	1.00 (0.84–1.20)	2503	1.25 (0.98–1.59)	2503	0.98 (0.70–1.35)	2503	0.03 (−0.02 to 0.07)
Secondary analysis								
Psychotic experiences^f^	2531	1.13 (0.94–1.35)	2513	0.80 (0.63–1.01)	2513	1.02 (0.74–1.41)	2515	0.06 (0.01–0.10)
Depression	2531	1.08 (0.90–1.30)	2513	1.02 (0.81–1.29)	2513	1.53 (1.09–2.16)	2515	0.10 (0.06–0.15)***
Anxiety	2531	1.07 (0.89–1.29)	2513	1.02 (0.80–1.30)	2513	1.22 (0.88–1.69)	2515	0.09 (0.04–0.13)***
Neuroticism	2531	1.17 (0.97–1.40)	2513	0.98 (0.78–1.24)	2513	1.01 (0.74–1.39)	2515	0.11 (0.06–0.15)***
Autism	2531	0.90 (0.75–1.08)	2513	1.08 (0.86–1.37)	2513	0.94 (0.68–1.29)	2515	0.09 (0.05–0.13)***

OR, odds ratio (1.00 = no effect); *β*, beta-coefficient (0 = no effect).

a. Logistic regression for interviewer-rated experiences/symptoms and linear regression for self-rated negative symptoms, using PRS standardised scores based on a Genome-Wide Association Study discovery sample threshold of *P* = 0.05, restricted to White ethnicity, adjusted for gender and 10 population genetic ancestry principal components.

b. Present if hallucination or delusion experience assessed by interviewer as being present at 12–24 years.

c. Present if reduced interaction/speech or restricted affect observed at interview at 24 years.

d. Present if incoherent speech or odd/inappropriate behaviour observed at interview at 24 years.

e. Sum score (0–30) of self-rated negative symptom questions from Community Assessment of Psychic Experiences (CAPE) questionnaire at 24 years (scores square-root transformed because of positive skew).

f. Psychotic experiences in middle-aged/older adults.

***P < 0.001, two-tailed.

The results of secondary analysis of the narrow Pos, Neg and Dis dimensions are shown in Supplementary Tables 2–5, and associations were also non-significant.

### Associations between individual psychotic experiences and schizophrenia PRS

The results of this secondary analysis are shown in Supplementary Table 7; again these were non-significant.

### Associations between psychotic experience dimensions and other PRSs

The results of this secondary analysis are shown in Table 2, with further details in Supplementary Tables 2–5 and Supplementary Figs 1 and 2.

None of the broad or narrow Pos, Neg or Dis dimensions showed associations at *P* < 0.001.

Higher self-rated Neg scores were associated (*P* < 0.001) with higher depression, anxiety, neuroticism and autism PRSs. However, effect sizes were too small (*β* = 0.09–0.11) to be clinically actionable at present.

### Associations between psychotic experience dimensions and other variables

The results of this secondary analysis are shown in Supplementary Tables 2–5.

Broad Pos was associated (*P* < 0.001) with lower IQ at 8 years, lower mental well-being at 23 years, depressive and generalised anxiety disorders at 24 years and lower social rapport at 24 years.

Broad Neg and Dis were associated with lower social rapport at 24 years.

Self-rated Neg was associated with male gender and lower mental well-being at 23 years, depressive and generalised anxiety disorders at 24 years and lower social rapport at 24 years.

### Associations between psychotic disorder and PRSs and other variables

The results of this secondary analysis are shown in Supplementary Table 8.

Psychotic disorder was not associated with schizophrenia PRS (odds ratio 1.10 [95% CI 0.85–1.42], *P* = 0.483). It was not associated with other PRSs at *P* < 0.001, but was associated with lower mental well-being at 23 years, depressive and generalised anxiety disorders at 24 years and lower social rapport at 24 years.

### Additional secondary analysis exploring subdimensions of positive psychotic experiences and CAPE questions

Having obtained the above results, we conducted an additional secondary exploratory factor analysis of the 12 types of hallucination and delusion experience in the PLIKS interview, to identify further substructure among these experiences (see Supplementary Method and Results and Supplementary Table 9 for further details). We identified two factors: the first (labelled first-rank delusions) comprised delusions of control, and thought insertion, withdrawal and broadcast; the second (labelled paranoid) comprised auditory and visual hallucinations, delusions of being spied on and delusions of persecution.

On logistic regression analysis, the presence of first-rank delusion experiences was nominally associated with higher schizophrenia PRS (*n* = 2535, odds ratio 7.35 [95% CI 2.10–25.77], *P* = 0.002), but the presence of paranoid experiences was not (*n* = 2526, odds ratio 1.01 [95% CI 0.84–1.22], *P* = 0.929) (Supplementary Fig. 3).

We also conducted exploratory factor analysis of the ten individual CAPE questions but, because the first component dominated, we did not conduct further analysis.

## Discussion

Against expectation, none of the primary psychotic experience dimensions was associated with schizophrenia PRS.

### Interviewer-rated positive dimension

In contrast to the lack of association for the current interviewer-rated positive psychotic experience dimension, positive schizotypal experiences, as assessed by interview, have been associated with higher schizophrenia PRS derived from an earlier GWAS^26^ (*n* = 523). The reasons for variation in results are not clear, but the schizotypy study had a broader age range (inclusion criteria 16–50 years, mean age 31.1 years (s.d. 10.7)) and, in addition to psychotic experiences, included, for example, magical ideation and derealisation.

In ALSPAC, there was no association between positive psychotic experiences from interviews at 12 or 18 years and an earlier schizophrenia PRS,^13^ but there was an association for self-rated positive psychotic experiences at 16 years.^14^ In addition to differences in assessment method (self-rated versus interview) and time frame, there were some differences in participants between the 16- and 24-year assessments, but we did not have the 16-year PLIKS questionnaire data to investigate further. However, consistent with the current study, there was no association with schizophrenia PRS in a combined analysis of self-rated positive psychotic experiences in ALSPAC at 16 years and two adolescent twin samples.^23^ This is because participants without psychotic experiences often had a schizophrenia PRS similar to those with high psychotic experience scores.

There is an association between the presence of self-rated positive psychotic experiences and schizophrenia PRS in a larger sample of middle-/older-aged adults, but of small effect size (odds ratio 1.09 [95% CI 1.06–1.12]) and non-specific, showing similar associations with other PRSs.^24^

In clinical samples, most studies of probands with schizophrenia show a lack of association between the number/severity of positive symptoms and schizophrenia PRS,^4,6,16,17^ but there is an association in bipolar spectrum disorders, especially for mood-incongruent psychotic symptoms.^19,20^ This may be because of greater variation in positive symptoms and schizophrenia liability within bipolar than schizophrenia samples.^15^

Some studies of broad psychosis spectrum samples also show associations between the number/severity of positive symptoms and schizophrenia PRS,^5,18^ but not consistently.^21,22^ Considerable methodological heterogeneity makes comparison of these studies difficult.

In the current study, positive psychotic experiences were associated with lower mental well-being, depressive and anxiety disorders and lower childhood IQ, consistent with other studies.^34,36,37^ Positive psychotic experiences have also been associated with other risk factors, including childhood adversity and cannabis use.^36,37^

There were no associations of global hallucination or delusion experiences, paranoid experiences or psychotic disorder with schizophrenia PRS. First-rank delusion experiences were nominally associated, but important caveats are the very small number of participants with these experiences (*n* = 5 (0.2%) with PRS), a wide confidence interval and the risk of a false positive result from multiple testing in this additional secondary analysis.

The concept of first-rank symptoms was devised with a view to identifying relatively specific symptoms of schizophrenia, but these have been de-emphasised in ICD-11^38^ and DSM-5-TR,^39^ for reasons including that they are also found in other conditions such as mania. However, as noted above, there is evidence that, within bipolar spectrum disorders, mood-incongruent psychotic symptoms, which include first-rank symptoms, are associated with higher schizophrenia PRS.^19^

In view of this, the exploratory and highly provisional association between first-rank delusions and schizophrenia PRS is interesting, but requires independent confirmation.

### Interviewer-rated negative dimension

In contrast to the current findings, parent-rated negative symptoms were associated with higher scores on an earlier schizophrenia PRS in the combined analysis of ALSPAC assessments at 16 years and two adolescent twin samples.^23^

Associations have also been found between negative/disorganised schizotypal features, as assessed by interview, and schizophrenia PRS,^17,26^ but it is unclear whether it was the negative, disorganised or both phenomena that contributed to the association.

In clinical samples of schizophrenia and other psychotic disorders, negative symptom dimensions have frequently been associated with genetic loading for schizophrenia, including PRS.^16^ Again there is a caveat that the negative dimensions may include disorganised symptoms. Some studies that attempted to disaggregate these symptoms,^4^ or examined the independence of their associations,^6^ found that the association was predominantly with disorganised symptoms.

A further complication in clinical samples is that negative symptoms could be markers of primary developmental aetiological processes and/or secondary to having a psychotic disorder – for example, to the effects of positive symptoms, medication or social isolation.

In a study of adolescent twins at 16 years, i.e. prior to the main age at onset range of psychotic disorders, parent-rated negative symptoms (presumed to be predominantly primary symptoms) were associated with male gender, lower parental socioeconomic status, cumulative pre/perinatal complications, lower cognitive functioning in childhood and lower educational attainment.^37^ The association with male gender is also often found in clinical samples of psychotic disorders, along with indices of relatively severe chronic illness^8^ and cognitive impairment.^40^

In the current study there was an association with lower social rapport, which could result in social isolation, and a nominal association with lower childhood IQ; otherwise, the pattern of associations was not strikingly similar to those in adolescent population or in clinical studies. It is unclear, therefore, how much the one-off interviewer observations at 24 years are able to identify the same phenotypes as in the other studies.

### Interviewer-rated disorganised dimension

Regarding the interviewer-rated disorganised dimension, there has been little comparable research in general population samples. As mentioned above, associations have been found between interview-based negative/disorganised schizotypal phenotypes and higher schizophrenia PRS,^17,26^ but it is unclear which phenomena contributed to the association.

In clinical schizophrenia and psychosis samples, there is relatively consistent evidence that the disorganised symptom dimension has substantial heritability^9,10^ and is associated with higher schizophrenia PRS.^4,6,17^

In population samples there are investigations of self-rated cognitive disorganisation, which shows association with schizophrenia PRS.^23^ However, because there is little correlation between self- and observer-rated disorganised symptoms in clinical samples,^41^ the relevance of the self-rated cognitive disorganisation studies to the current interviewer-rated disorganised dimension is unclear.

In clinical samples, the disorganised symptom dimension is associated with lower cognitive functioning,^40^ including lower premorbid IQ,^8^ which is consistent with the nominal association with lower childhood IQ in the current study. In addition, the apparently odd speech and behaviour of people with disorganised symptoms is consistent with the association with lower social rapport in the current study.

Nevertheless, the lack of association with schizophrenia PRS in the current study raises the question of how similar or different are the aetiological processes underlying interviewer-observed disorganised phenomena at a single time point in the general population compared with clinically observed disorganised symptoms, which are often clearest during acute psychotic episodes.

### Self-rated negative dimension

In contrast to the results for the current self-rated negative dimension based on the CAPE questionnaire at 24 years, there was an association between the self-rated negative dimension based on CAPE at 16 years in ALSPAC and an earlier schizophrenia PRS.^13,14^ As with the positive dimension, we did not have the CAPE data at 16 years to further investigate this difference.

In other general population studies of the CAPE self-rated negative dimension, results have also been inconsistent, in that both association^5^ and absence of association^26^ with schizophrenia PRS have been found.

In contrast to the lack of association with schizophrenia PRS in the current study, there were substantive associations with depression, anxiety, neuroticism and autism PRSs, although not of sufficient effect size to be clinically actionable. Association with neuroticism PRS was also found at 16 years in ALSPAC.^14^ Self-rated negative schizotypal experiences have also been associated with depression and neuroticism PRSs in another general population sample.^25^

Consistent with the PRS results, higher scores on the self-rated negative dimension were associated with the presence of depressive and generalised anxiety disorders, as well as with lower mental well-being and social rapport, at 24 years.

There was no correlation between interviewer- and self-rated negative dimensions in the current study, which suggests that they may be assessing phenotypes with notably different aetiologies. Low correlations between self- and observer-rated negative symptoms have also been found in some clinical samples.^41^

In summary, self-rated negative symptoms were not associated with schizophrenia PRS in the current study, and show inconsistent associations in other studies. They may be influenced by genetic risk variants for other disorders, but confirmation in further independent samples would be valuable.

### Strengths and limitations

To our knowledge, this is the first birth cohort study to assess distinct positive, negative and disorganised psychotic experience dimensions, including the use of interviewer observations for negative and disorganised symptoms, and additionally using self-rated negative symptoms, in large sample sizes of 2500+ at 24 years.

Despite assessment around the peak age at onset of schizophrenia,^7^ clearly present interview-based psychotic experiences remained infrequent. This limited statistical power and exploration of the dimension construct, especially regarding the gradient of scores including co-occurring experiences.

Results could have differed if we had used other definitions of psychotic experience dimensions. We explored the use of factor scores, but were concerned about the stability of factors given the low frequency of some experiences, which also precluded splitting the sample for exploratory and confirmatory factor analysis. Furthermore, the use of simple phenotypes based on whether or not experiences were clearly present simplified interpretation and comparison with similar definitions in clinical samples.^8^ These simple phenotypes also show similar levels of familial aggregation to phenotypes based on factor scores in clinical samples.^2,3^

The results could also have differed if we had used other approaches to generating PRSs. However, lack of association of positive psychotic experiences and negative symptoms with schizophrenia PRS was also found in a larger sample of adolescent twins using other PRS approaches.^37,42^ Additionally, the approach in the current study facilitated comparison with other studies, most of which also used the GWAS discovery sample *P*-value threshold approach.

The sample size was substantial, but combination with other samples in meta-analyses may be required to detect associations of small effect.

There were considerable missing data, due to participant attrition between inception and 24 years. Individuals were more likely to have missing data at 24 years if they were male, had greater socioeconomic disadvantage or had had more severe psychotic experiences at 18 years.^12^ Observed and imputed estimates are very similar for the frequency of any definite positive psychotic experience and psychotic disorder at 12–24 years,^12^ but biased estimates are still possible, including because of data not missing at random.

There is also an association between non-participation and higher schizophrenia PRS in ALSPAC, leading to reduced power for analyses of schizophrenia and genetically related psychiatric phenotypes, and potentially biased estimates, even with imputation due to data not missing at random.^43^

Inverse probability weighting may also be used to adjust for missing data, but issues include the assumption that, for example (if being male is a predictor of missing data), male non-participants would have similar associations to male participants.^44^

The genetic analysis was confined to associations with PRS and did not include rarer schizophrenia risk variants.^15^

The sample was predominantly of White European ancestry, and exclusively so for the PRS analyses, which minimised the risk of confounding by population stratification. However, further studies in more ethnically diverse samples would be valuable, which is a major focus of the Psychiatric Genomics Consortium (https://pgc.unc.edu).

The results of this study suggest that positive, negative and disorganised experience/symptom dimensions, as defined here and assessed in this general population cohort of young adults, are not strongly associated with polygenic liability to schizophrenia, although true associations hidden by methodological constraints are not excluded, e.g. due to small effect sizes, missing data, low phenotype base rates or misclassification, or PRS limitations.

Positive psychotic experiences may be (modestly and non-specifically) influenced by commonly occurring risk alleles for schizophrenia, with PRS associations seen more from middle age onwards, as people with high schizophrenia liability but no lifetime psychotic experiences become fewer over time. Future research in ALSPAC and other samples as participants age may clarify whether this is the case.

In clinical samples, schizophrenia PRS is most consistently associated with disorganised and/or negative symptoms. The interviewer-rated negative and disorganised symptoms in the current study probably have notably different aetiologies. Also, the self-rated negative symptoms may be better indicators of social withdrawal/low motivation due to polygenic liability to depression, anxiety, neuroticism or autism than schizophrenia.

The highly provisional secondary finding, that first-rank delusions might be indicators of higher polygenic liability to schizophrenia, is of interest, but needs confirmation in other samples where information on first-rank symptoms is available before substantive conclusions can be drawn.

## Supporting information

Cardno et al. supplementary material 1 Cardno et al. supplementary material

Cardno et al. supplementary material 2 Cardno et al. supplementary material

Cardno et al. supplementary material 3 Cardno et al. supplementary material

Cardno et al. supplementary material 4 Cardno et al. supplementary material

Cardno et al. supplementary material 5 Cardno et al. supplementary material

## Data Availability

The Avon Longitudinal Study of Parents and Children (ALSPAC) study website contains details of all the data that are available, through a fully searchable data dictionary and variable search tool (http://www.bristol.ac.uk/alspac/researchers/our-data/). Access to ALSPAC data is through a system of managed open access.

## Supplementary material

The supplementary material is available online at https://doi.org/10.1192/bjo.2025.10825
