# Supplementary material for: Associations between psychotic experience dimensions and polygenic liability to schizophrenia in a longitudinal birth cohort
Source: BJPsych Open. 2025 Sep 8;11(5):e197. doi: 10.1192/bjo.2025.10825 (PMC12451531; doi:10.1192/bjo.2025.10825)
Supplement: Cardno et al. supplementary material 1 — Cardno et al. supplementary material [file S2056472425108259sup001.docx]

**Associations Between Psychotic Experience Dimensions and Polygenic Liability to Schizophrenia in a Longitudinal Birth Cohort**

Alastair G Cardno, Hein Heuvelman, Sophie E Legge, James T R Walters, Stanley Zammit, Hannah J Jones

**Supplementary Method and Results**

Further information on the PLIKS interview at age 24y^1^

The semi-structured Psychosis-Like Symptoms Interview includes 12 core questions eliciting key psychotic experiences: hallucinations (visual and auditory), delusions (spied on, persecution, thoughts read, reference, control, grandiosity, and other), and experiences of thought interference (broadcasting, insertion, and withdrawal). Questions about each experience started with a structured stem question asking if the participant had ever had that experience since age 12. Participants endorsing “yes” or “maybe” responses (referred to as “self-reported experiences”) were then cross-questioned to establish whether the experience was psychotic (referred to as “interview-rated experiences”). Coding of psychotic experiences followed glossary definitions and rating rules for the Schedules for Clinical Assessment in Neuropsychiatry (SCAN).^2^ Interviewers rated psychotic experiences as not present, suspected, or definitely present. Unclear responses after probing were “rated down,” and items were rated as definite only when an example that clearly met the rating rules for the Schedules for Clinical Assessment in Neuropsychiatry was provided.

The interviewers were psychology graduates trained in using the PLIKS interview, and blind to previous PLIKS assessments. Each interview was conducted by a single interviewer. Interviewers had to score >0.9 agreement with ‘gold-standard’ ratings on 2 audio-recorded interviews before they were able to start collecting data for the study. At regular intervals, a psychiatrist rated samples of recorded interviews to ensure that the interviewers were rating experiences correctly.

To test inter-rater reliability, interviewers audio-recorded interviews at approximately 9, 18, and 24 months after the start of the age-24 clinic (15 raters; 70 interviews). The kappa statistic for the rating of any psychotic experience rated as suspected or definitely present was 0.61 (n=402), and the ICC was 0.81 (95% CI 0.68, 0.89). Test-retest reliability was undertaken using data from 103 individuals who were re-interviewed after approximately 5 weeks (mean 43 days, SD 12.5 days; range 27 to 77 days). Only 20 individuals were interviewed by the same interviewer on both occasions, and thus our test-retest reliability estimates are likely to be under-estimates. Test-retest agreement for any psychotic experience was 94% (kappa 0.64; ICC 0.9, 95%CI 0.83, 0.95).

After conducting the PLIKS interview, the interviewers also made ratings of observed variables, including level of interaction, range of affect, behaviour, and speech coherence, each rated on 1-5 scales (see Supplementary file - Observer ratings from interview - for symptom rating guide). Scores of 1 or 2 indicated a negative or disorganised symptom being clearly present. The observed ratings were included in the training of interviewers but formal assessments of reliability were not conducted.

The PLIKS interview allowed positive psychotic experiences, negative symptoms and disorganised symptoms to be assessed in as similar a way as possible to clinical studies of psychotic symptom dimensions, which often employ semi-structured interviews such as the SCAN. Clinical studies also normally review clinical case records, which were not available in the current study.

Further information on the CAPE questionnaire for self-rated negative symptoms^3^

The presence of negative symptoms was assessed using 10 questions based on items from the Community Assessment of Psychic Experiences (CAPE) self-report questionnaire^4,5^ at age 24 years (see Supplementary file - CAPE questions - for the questions used). These questions covered a range of negative symptom domains including apathy, anergia, shyness, asociality and attention to appearance. The questions measured the frequency of occurrence of negative symptoms using a 4-point scale (0: never, 1: sometimes, 2: often, 3: always) relating to the past month.

The PLIKS interviewer-rated and CAPE self-rated negative symptom assessments complemented each other. The PLIKS assessment allowed observation of reduced emotional expression aspects of negative symptoms, while the CAPE assessment included reduced motivation and socialisation aspects of negative symptoms. We also included the CAPE self-rated negative symptoms as these were associated with higher schizophrenia PRS in ALSPAC at 16y.^3,6^

Genetic data^6^

Genetic data were acquired using the Illumina HumanHap550 quad genome-wide single nucleotide polymorphism (SNP) genotyping platform from 9912 participants. Individuals were excluded from further analysis based on gender mismatches, minimal or excessive heterozygosity, disproportionate levels of individual missingness (>3%), evidence of cryptic relatedness (>10% of alleles identical by descent), and being of non-European ancestry (assessed by multidimensional scaling analysis including HapMap 2 individuals). SNPs with a minor allele frequency (MAF) of < 1%, Impute2 information quality metric of < 0.8, a call rate of < 95% or evidence for violations of Hardy-Weinberg equilibrium (P-value < 5e^-7^) were removed. Imputation of the target data was performed using Impute V2.2.2 against the 1000 genomes reference panel (Phase 1, Version 3; all polymorphic SNPs excluding singletons), using 2186 reference haplotypes (including non-Europeans). Following quality control assessment and imputation and restricting to 1 young person per family, genetic data was available for 7856 ALSPAC individuals.

Polygenic risk scores^6^

Polygenic risk scores (PRSs) were constructed using GWAS summary statistics from discovery studies for schizophrenia,^7^ psychotic experiences in middle-aged/older adults,^8^ depression,^9^ anxiety,^10^ neuroticism,^11^ and autism.^12^

SNPs were excluded from the analysis if they had a minor allele frequency less than 0.01, imputation quality less than 0.8, or if there was allelic mismatch between samples (alleles reported by the discovery study not matching alleles in the ALSPAC sample). Due to the high linkage disequilibrium (LD) within the extended major histocompatibility complex (MHC; chromosome 6: 25-34Mb) only a single SNP was included to represent this region. Remaining SNPs were then further pruned for LD using the PLINK (v1.07)^13^ ‘clump’ command to retain SNPs with a trait association *P*-value ≤ 0.5 and *r*^2^ < 0.25 within 500kb windows.

Polygenic scores were calculated for each ALSPAC individual using PLINK (v1.07)^13^ by summing the number of risk alleles for each SNP (0, 1 or 2) weighted by the logarithm of its discovery sample odds ratio (OR) for binary traits (schizophrenia, depression, anxiey and autism) or by the beta for continuous traits (neuroticism). Our analysis used scores generated from a list of SNPs with a GWAS training-set *P*-value threshold (*P*_T_) ≤ 0.05.

This is the optimal threshold for distinguishing between clinical cases and controls. It does not necessarily extrapolate to general population samples of adolescents and young adults. However, previous ALSPAC studies that have found PRS associations with psychotic experiences have in practice done so at this threshold as well.^3,6^

Also, PRS approaches are based on an additive model of genetic inheritance. While this is regarded as a reasonable model to employ on the basis of current knowledge, it should be noted that the detailed genetic architecture of variants that causally influence psychotic symptoms and experiences is not yet established.

Secondary analysis of demographics, developmental risk factors, affective disorders, quality of life, social rapport, and psychotic disorder

To give context and possible insights into the primary results, we also conducted associations of the psychotic experience dimensions with the following demographics, developmental risk factors, affective disorders, quality of life and social rapport variables:

Sex; ethnicity; mother's socioeconomic status and education level, and her partner's socioeconomic status, when participant born.

Maternal smoking at start of pregnancy^14^ (no. times/day).

Birthweight^15^ (kg).

Childhood cognitive functioning^16,17^ – full-scale Wechsler Intelligence Scale for Children (WISC) IQ^18^ at 8y.

Mental wellbeing - The Warwick-Edinburgh Mental Well-being Scale (WEMWBS) questionnaire composite score^19^ assessed at 23y.

Moderate or severe depressive episode - ICD-10 diagnosis at 24y, assessed from interview.

Generalised anxiety disorder - ICD-10 diagnosis at 24y, assessed from interview.

Social rapport - PLIKS interviewer observed rating at 24y (1-5 scale: 1=no connection to 5=very good rapport).

Family history of schizophrenia - present/absent in a first or second degree relative.

Each of the variables has been previously associated with psychotic symptoms or experiences, or plausibly could be, and the aims of analysing them were to better understand the psychotic experience dimensions in terms of the pattern of their associations; to compare the pattern of associations found here with other studies; and to check the consistency of associations, e.g. whether a psychotic experience dimension associated with depression or anxiety PRS was also associated with depressive or anxiety disorders.

Finally, we analysed associations between presence/absence of psychotic disorder and the above PRSs and other demographic/risk factor variables using logistic regression analysis, in order to gain insights into differences in associations between psychotic symptoms and broad psychotic experiences. Psychotic disorder was defined as the presence of one or more positive psychotic experiences occurring between 12y and 24y, and accompanied by significant distress, functional impairment or help-seeking.^1^

Additional secondary analysis exploring sub-dimensions of PLIKS interview positive psychotic experiences and CAPE questions

After obtaining the planned primary and secondary results, we conducted the following additional secondary analysis in SPSS version 29 for Mac.

*Individual PLIKS interview positive psychotic experiences*

In view of the lack of association of the psychotic experience dimensions and individual global psychotic experiences with schizophrenia PRS, and because there was a range of experiences within the global hallucination and delusion experiences, we conducted an additional secondary exploratory factor analysis of each of the 12 types of hallucination and delusion experience in the PLIKS interview, in order to identify further substructure among these experiences. We restricted this further analysis to positive psychotic experiences as the interviewer-rated negative and disorganised symptoms were assessed in a different timeframe (observed at interview rather than reported to have occurred between 12y and 24y) and were not subdivided into a similar range of subtypes of experience.

We conducted a principal components analysis of the 12 types of hallucination and delusion experience, each rated 0-2 (absent/uncertain/present), in 3862 participants. The scree plot suggested two factors, which accounted for 37.9% of the variance. Orthogonal (varimax) and oblique (direct oblimin) principal components rotations showed a similar pattern of loadings. The factor loadings from the varimax rotation are shown in supplementary Table S9. The first factor (labelled first rank delusions) had relatively specific high loadings (>0.5) for delusions of control, and thought insertion, withdrawal and broadcast. The second factor (labelled paranoid) had relatively specific high loadings for auditory and visual hallucinations, delusions of being spied on and delusions of persecution. The Spearman correlation between the sum scores of the high loading symptoms on the two factors was 0.19. We have found factors similar to both of these in clinical samples when a range of individual positive symptoms are analysed.^20,21^

11 participants had at least one first rank delusion experience (0.3%) (5 of whom had PRS data (0.2%)) and 199 had at least one paranoid experience (5.1%) (117 of whom had PRS data (4.6%)). In view of the small number of participants with first rank delusion experiences, and in keeping with our general approach of focusing on simple phenotypes that are relatively easy to interpret, we defined the first rank delusions and paranoid dimensions as the presence of any of the four experiences with high loadings on the relevant factor.

*Individual CAPE self-rated negative symptom questions*

In view of the lack of association between self-rated global negative symptoms and schizophrenia PRS, we conducted an additional secondary exploratory factor analysis of the 10 individual CAPE questions.

We conducted a principal components analysis of the 10 individual CAPE questions, each rated 0-3 (never/sometimes/often/always), in 3823 participants. The first component dominated, accounting for 50.3% of the variance, and all 10 questions had high component loadings (>0.5), so we did not conduct further analysis of the CAPE questions.

**References**

1. Sullivan SA, Kounali D, Cannon M, David AS, Fletcher PC, Holmans P, et al. A population-based cohort study examining the incidence and impact of psychotic experiences from childhood to adulthood, and prediction of psychotic disorder. *Am J Psychiatry* 2020; **177**: 308-17.

2. Wing JK, Babor T, Brugha T, Burke J, Cooper JE, Giel R, et al. SCAN. Schedules for Clinical Assessment in Neuropsychiatry. *Arch Gen Psychiatry* 1990; **47**: 589-93.

3. Jones HJ, Stergiakouli E, Tansey KE, Hubbard L, Heron J, Cannon M, et al. Phenotypic manifestation of genetic risk for schizophrenia during adolescence in the general population. *JAMA Psychiatry* 2016; **73**: 221-28.

4. Konings M, Bak M, Hanssen M, van Os J, Krabbendam L. Validity and reliability of the CAPE: a self-report instrument for the measurement of psychotic experiences in the general population. *Acta Psychiatr Scand* 2006; **114**: 55-61.

5. Stefanis NC, Hanssen M, Smirnis NK, Avramopoulos DA, Evdokimidis IK, Stefanis CN, et al. Evidence that three dimensions of psychosis have a distribution in the general population. *Psycholl Med* 2002; **32**: 347-58.

6. Jones HJ, Heron J, Hammerton G, Stochl J, Jones PB, Cannon M, et al. Investigating the genetic architecture of general and specific psychopathology in adolescence. *Transl Psychiatry* 2018; **8**: 145.

7. Trubetskoy V, Pardiñas AF, Qi T, Panagiotaropoulou G, Awasthi S, Bigdeli TB, et al. Mapping genomic loci implicates genes and synaptic biology in schizophrenia. *Nature* 2022; **604**: 502-8.

8. Legge SE, Jones HJ, Kendall KM, Pardiñas AF, Menzies G, Bracher-Smith M, et al. Association of genetic liability to psychotic experiences with neuropsychotic disorders and traits. *JAMA Psychiatry* 2019; **76**: 1256-65.

9. Howard DM, Adams MJ, Clarke TK, Hafferty JD, Gibson J, Shirali M, et al. Genome-wide meta-analysis of depression identifies 102 independent variants and highlights the importance of the prefrontal brain regions. *Nat Neurosci* 2019; **22**: 343-52.

10. Purves KL, Coleman JRI, Meier SM, Rayner C, Davis KAS, Cheesman R, et al. A major role for common genetic variation in anxiety disorders. *Mol Psychiatry* 2020; **25**: 3292-303.

11. Luciano M, Hagenaars SP, Davies G, Hill WD, Clarke TK, Shirali M, et al. Association analysis in over 329,000 individuals identifies 116 independent variants influencing neuroticism. *Nat Genet* 2018; **50**: 6-11.

12. Grove J, Ripke S, Als TD, Mattheisen M, Walters RK, Won H, et al. Identification of common genetic risk variants for autism spectrum disorder. *Nat Genet* 2019; **51**: 431-44.

13. Purcell S, Neale B, Todd-Brown K, Thomas L, Ferreira MAR, Bender D, et al. PLINK: a tool set for whole-genome association and population-based linkage analyses. *Am J Hum Genet* 2007; **81**: 559-75.

14. Stathopoulou A, Beratis IN, Beratis S. Prenatal tobacco smoke exposure, risk of schizophrenia, and severity of positive/negative symptoms. *Schizophr Res* 2013; **148**: 105-10.

15. Cannon M, Jones PB, Murray RM. Obstetric complications and schizophrenia: historical and meta-analytic review. *Am J Psychiatry* 2002; **159**: 1080-92.

16. Mollon J, David AS, Zammit S, Lewis G, Reichenberg A. Course of cognitive development from infancy to early adulthood in the psychosis spectrum. *JAMA Psychiatry* 2018; **75**: 270-9.

17. Woodberry KA, Giuliano AJ, Seidman LJ. Premorbid IQ in schizophrenia: a meta-analytic review. *Am J Psychiatry* 2008; **165**: 579-87.

18. Wechsler D, Golombok J, Rust J. *Wechsler Intelligence Scale for Children—Third Edition UK (WISC-IIIUK) Manual.* London: The Psychological Corporation, 1992.

19. Tennant R, Hiller L, Fishwick R, Platt S, Joseph S, Weich S, et al. The Warwick-Edinburgh Mental Well-being Scale (WEMWBS): development and UK validation. *Health Qual Life Outcomes* 2007; **5**: 63.

20. Cardno AG, Jones LA, Murphy KC, Asherson P, Scott LC, Williams J, et al. Factor analysis of schizophrenic symptoms using the OPCRIT checklist. *Schizophr Res* 1996; **22**: 233-9.

21. Cardno AG, Sham PC, Murray RM, McGuffin P. Twin study of symptom dimensions in psychoses. *Br J Psychiatry* 2001; **179**: 39-45.
