# Supplementary material for: Associations between psychotic experience dimensions and polygenic liability to schizophrenia in a longitudinal birth cohort
Source: BJPsych Open. 2025 Sep 8;11(5):e197. doi: 10.1192/bjo.2025.10825 (PMC12451531; doi:10.1192/bjo.2025.10825)
Supplement: Cardno et al. supplementary material 3 — Cardno et al. supplementary material [file S2056472425108259sup003.docx]

**Associations Between Psychotic Experience Dimensions and Polygenic Liability to Schizophrenia in a Longitudinal Birth Cohort**

Alastair G Cardno, Hein Heuvelman, Sophie E Legge, James T R Walters, Stanley Zammit, Hannah J Jones

**Supplementary Figures**

**Fig. S1 Association of interviewer-rated psychotic experience dimensions with schizophrenia and other polygenic risk scores**

Logistic regression analysis of interviewer-rated psychotic experience dimensions on polygenic risk scores, using PRS standardised scores based on a GWAS discovery sample threshold of pt0.05, restricted to white ethnicity, adjusted for sex and 10 population genetic ancestry principal components.

Positive psychotic experiences regarded as present if hallucination or delusion experience assessed by interviewer as present at 12-24y.

Negative symptoms regarded as present if reduced interaction/speech or restricted affect observed at interview at 24y.

Disorganised symptoms regarded as present if incoherent speech or odd/inappropriate behaviour observed at interview at 24y.

All error bars represent the 95% confidence interval of the odds ratio. Dashed line represents a null model (values > 1 indicate increased risk and values < 1 indicate reduced risk).

**Fig. S2 Association of self-rated negative symptom dimension with schizophrenia and other polygenic risk scores**

Linear regression analysis of self-rated negative symptoms on polygenic risk scores, using PRS standardised scores based on a GWAS discovery sample threshold of pt0.05, restricted to white ethnicity, adjusted for sex and 10 population genetic ancestry principal components.

Self-rated negative symptoms score is the sum score (0-30) of self-rated negative symptom questions from Community Assessment of Psychic Experiences (CAPE) questionnaire at 24y (scores square-root transformed because of positive skew).

All error bars represent the 95% confidence interval of the beta value. Dashed line represents a null model (values > 0 indicate increased risk and values < 0 indicate reduced risk).

**Fig. S3 Association of interviewer-rated positive psychotic experience sub-dimensions with schizophrenia polygenic risk score**

Logistic regression analysis of interviewer-rated positive psychotic experience sub-dimensions on schizophrenia polygenic risk score, using PRS standardised scores based on a GWAS discovery sample threshold of pt0.05, restricted to white ethnicity, adjusted for sex and 10 population genetic ancestry principal components.

First rank delusion experiences regarded as present if delusions of control or thought insertion, withdrawal or broadcast assessed by interviewer as present at 12-24y.

Paranoid experiences regarded as present if auditory hallucinations, visual hallucinations, delusions of being spied on or delusions of persecution assessed by interviewer as present at 12-24y.

All error bars represent the 95% confidence interval of the odds ratio. Dashed line represents a null model (values > 1 indicate increased risk and values < 1 indicate reduced risk).

Figures made in R version 4.5.0 (https://cran.r-project.org).
