# Supplementary material for: Associations between psychotic experience dimensions and polygenic liability to schizophrenia in a longitudinal birth cohort
Source: BJPsych Open. 2025 Sep 8;11(5):e197. doi: 10.1192/bjo.2025.10825 (PMC12451531; doi:10.1192/bjo.2025.10825)
Supplement: Cardno et al. supplementary material 4 — Cardno et al. supplementary material [file S2056472425108259sup004.docx]

**Associations Between Psychotic Experience Dimensions and Polygenic Liability to Schizophrenia in a Longitudinal Birth Cohort**

Alastair G Cardno, Hein Heuvelman, Sophie E Legge, James T R Walters, Stanley Zammit, Hannah J Jones

**Observed Ratings from PLIKS Interview**

**Negative symptoms**

Level of interaction (score 1 or 2 = Reduced interaction/speech clearly present)

| **1** | **2** | **3** | **4** | **5** |
| --- | --- | --- | --- | --- |
| Little attempt to interact during interview  Withdrawn, minimal eye contact, answers as brief as possible (if at all) | Clearly reduced interaction compared to most other people  Answers often brief and unembellished | Some hesitation in initiating interaction  Answers sometimes brief and unembellished | Initiates interactions well  Occasional instances of hesitation with initiating or continuing conversation | Initiates interactions very readily  Forthcoming with answers throughout |

Range of affect (score 1 or 2 = Restricted affect clearly present)

| **1** | **2** | **3** | **4** | **5** |
| --- | --- | --- | --- | --- |
| Very blunted  Face/voice expressionless  No change in emotional response when discussing positive and/or negative experiences | Clearly reduced range of affect | Some limitation in range of emotion expressed apparent  Facial expression and tone do not really convey emotions appropriate to experience discussed | Appropriate range of emotions though mild restriction noted occasionally | Full and appropriate range of emotions when discussing positive and/or negative experiences |

**Disorganised symptoms**

Behaviour (score 1 or 2 = Odd/inappropriate behaviour clearly present)

| **1** | **2** | **3** | **4** | **5** |
| --- | --- | --- | --- | --- |
| Very strange or unconventional behaviour or appearance | Behaviour that is quite noticeably different to that for most other participants | Somewhat odd or inappropriate behaviour at times  Somewhat different to that for most other participants | Behaviour very appropriate overall  Occasional instances where not (eg slightly over-familiar or immature) | Behaviour entirely appropriate to situation |

Speech coherence (score 1 or 2 = Incoherent speech clearly present)

Note to interviewers: this is rating of connections between thoughts and NOT of motor ability/mumbling etc.

| **1** | **2** | **3** | **4** | **5** |
| --- | --- | --- | --- | --- |
| Very muddled or incoherent  Very difficult to understand | Many examples of muddled thinking  Often difficult to make sense of responses | Sometimes vague, irrelevant, or going off track  Occasional difficulty making sense of response | Mostly very coherent  Instances where responses are vague / do not directly address question asked | Always coherent and relevant  No difficulty in understanding any responses made by participant |

**Questions Used to Create the Self-Rated Negative Symptoms Measure in ALSPAC^a^**

Have you felt that you are not much of a talker when you are chatting with other people?

Have you felt that you experience few or no emotions at important events, such as on your birthday?

Have you felt that you are lacking in motivation when you have to do things?

Have you felt that you are spending all your days doing nothing?

Have you felt that you are lacking 'get up and go'?

Have you felt that you have only a few hobbies or interests?

Have you felt that you have no interest to be with other people?

Have you felt that you are not a very lively person?

Have you felt that you are neglecting your appearance or personal hygiene?

Have you felt that you can never get things done?

a. Based on questions from the Community Assessment of Psychic Experiences (CAPE) self-report questionnaire.
