# Supplementary material for: Associations between psychotic experience dimensions and polygenic liability to schizophrenia in a longitudinal birth cohort
Source: BJPsych Open. 2025 Sep 8;11(5):e197. doi: 10.1192/bjo.2025.10825 (PMC12451531; doi:10.1192/bjo.2025.10825)
Supplement: Cardno et al. supplementary material 5 — Cardno et al. supplementary material [file S2056472425108259sup005.docx]

**Associations Between Psychotic Experience Dimensions and Polygenic Liability to Schizophrenia in a Longitudinal Birth Cohort**

Alastair G Cardno, Hein Heuvelman, Sophie E Legge, James T R Walters, Stanley Zammit, Hannah J Jones

**Supplementary Tables**

| Table S1. Spearman inter-correlations of 0-2 interviewer-rated psychotic experience/symptom scores and 0-30 self-rated negative symptoms | | | |
| --- | --- | --- | --- |
|  | Correlation | | |
|  | Positive | Negative | Disorganised |
| Positive | - | r_s_=0.032  p=0.047  n=3804 | r_s_=0.065  p<0.001  n=3805 |
| Negative | - | - | r_s_=0.360  p<0.001  n=3823 |
| Disorganised | - | - | - |
|  |  |  |  |
| Self-rated negative | r_s_=0.165  p<0.001  n=3699 | r_s_=0.024  p=0.153  n=3664 | r_s_=0.043  p=0.009  n=3667 |
|  |  |  |  |

| Table S2. Logistic regression analysis of broad and narrow positive dimension on demographics, developmental risk factors, affective disorders, quality of life and social rapport variables^a^ | | | | | |
| --- | --- | --- | --- | --- | --- |
| Independent variable | n | Broad positive dimension 12-24y  (score 0 vs 1-2) | | Narrow positive dimension 12-24y  (score 0-1 vs 2) | |
|  |  | OR (95%CI) | p-value | OR (95%CI) | p-value |
| Male sex | 3860 | 0.79 (0.59 to 1.06) | 0.117 | 0.62 (0.29 to 1.34) | 0.228 |
| Non-white ethnicity | 3459 | 0.46 (0.11 to 1.88) | 0.279 | 1.49 (0.20 to 11.05) | 0.699 |
|  |  |  |  |  |  |
| Mother’s highest educational qualification (CSE to degree) | 3462 | 0.84 (0.74 to 0.95) | 0.006** | 0.83 (0.62 to 1.11) | 0.207 |
| Social class at birth based on mother’s occupation (I [Professional] to V [Unskilled]) | 3089 | 1.15 (1.01 to 1.31) | 0.036* | 1.06 (0.76 to 1.46) | 0.746 |
| Social class at birth based on partner’s occupation (I [Professional] to V [Unskilled]) | 2852 | 1.09 (0.95 to 1.26) | 0.197 | 1.14 (0.82 to 1.59) | 0.446 |
|  |  |  |  |  |  |
| Maternal smoking at start of pregnancy (no. times/day) | 2787 | 1.03 (1.01 to 1.05) | 0.001** | 1.00 (0.95 to 1.06) | 0.964 |
| Birthweight (grams)  [standardised] | 3533 | 1.06 (0.90 to 1.25) | 0.461 | 1.06 (0.70 to 1.60) | 0.776 |
| WISC 8y (total IQ score) | 3104 | 0.98 (0.97 to 0.99) | 0.0002*** | 0.97 (0.949 to 0.997) | 0.029* |
|  |  |  |  |  |  |
| Mental wellbeing 23y (WEMWBS composite score) | 2777 | 0.94 (0.92 to 0.96) | 3.64x10^-12^*** | 0.90 (0.87 to 0.94) | 0.000005*** |
| Moderate or severe depressive episode 24y (ICD-10 diagnosis) | 3821 | 4.24 (2.99 to 6.01) | 4.53x10^-16^*** | 3.78 (1.68 to 8.51) | 0.001** |
| Generalised anxiety disorder 24y (ICD-10 diagnosis) | 3811 | 3.53 (2.52 to 4.93) | 1.61x10^-13^*** | 5.56 (2.68 to 11.54) | 0.000004*** |
| Social rapport 24y  (interviewer-rated: 1=no connection to 5=very good rapport) | 3804 | 0.72 (0.61 to 0.86) | 0.0002*** | 0.50 (0.34 to 0.73) | 0.0003*** |
|  |  |  |  |  |  |
| Family history of schizophrenia (1^st^ or 2^nd^ degree relatives) | 2716 | 1.31 (0.56 to 3.06) | 0.528 | 1.16 (0.16 to 8.65) | 0.886 |
| Schizophrenia PRS | 2521 | 1.00 (0.84 to 1.20) | 0.987 | 1.12 (0.70 to 1.78) | 0.639 |
| Psychotic experiences PRS (middle-older aged adults) | 2531 | 1.13 (0.94 to 1.35) | 0.207 | 1.35 (0.84 to 2.18) | 0.213 |
| Depression PRS | 2531 | 1.08 (0.90 to 1.30) | 0.386 | 0.88 (0.55 to 1.41) | 0.601 |
| Anxiety PRS | 2531 | 1.07 (0.89 to 1.29) | 0.461 | 0.85 (0.53 to 1.38) | 0.511 |
| Neuroticism PRS | 2531 | 1.17 (0.97 to 1.40) | 0.097 | 0.64 (0.41 to 1.00) | 0.049* |
| Autism PRS | 2531 | 0.90 (0.75 to 1.08) | 0.249 | 0.77 (0.49 to 1.20) | 0.243 |
|  |  |  |  |  |  |
| OR, odds ratio; 95%CI, 95% confidence interval; CSE, Certificate of Secondary Education; WISC, Wechsler Intelligence Scale for Children; WEMWBS, Warwick-Edinburgh Mental Well-being Scale; PRS, polygenic risk score.  a. Adjusted for sex (except the analysis of sex). PRS analyses using PRS standardised scores based on a GWAS discovery sample threshold of pt0.05, restricted to white ethnicity, adjusted for sex and 10 population genetic ancestry principal components.  *p<0.05, **p<0.01, ***p<0.001, two-tailed. | | | | | |

| Table S3. Logistic regression analysis of broad and narrow negative dimension on demographics, developmental risk factors, affective disorders, quality of life and social rapport variables^a^ | | | | | |
| --- | --- | --- | --- | --- | --- |
| Independent variable | n | Broad negative dimension  (score 0 vs 1-2) | | Narrow negative dimension  (score 0-1 vs 2) | |
|  |  | OR (95%CI) | p-value | OR (95%CI) | p-value |
| Male sex | 3823 | 0.74 (0.50 to 1.08) | 0.113 | 0.62 (0.34 to 1.13) | 0.121 |
| Non-white ethnicity | 3431 | 3.17 (1.42 to 7.07) | 0.005** | 5.34 (2.06 to 13.90) | 0.001** |
|  |  |  |  |  |  |
| Mother’s highest educational qualification (CSE to degree) | 3435 | 0.98 (0.84 to 1.16) | 0.846 | 0.99 (0.78 to 1.27) | 0.959 |
| Social class at birth based on mother’s occupation (I [Professional] to V [Unskilled]) | 3062 | 0.96 (0.80 to 1.15) | 0.635 | 1.05 (0.82 to 1.35) | 0.691 |
| Social class at birth based on partner’s occupation (I [Professional] to V [Unskilled]) | 2827 | 0.94 (0.78 to 1.13) | 0.519 | 1.02 (0.77 to 1.35) | 0.893 |
|  |  |  |  |  |  |
| Maternal smoking at start of pregnancy (no. times/day) | 2762 | 1.00 (0.97 to 1.03) | 0.864 | 1.00 (0.95 to 1.04) | 0.873 |
| Birthweight (grams)  [standardised] | 3501 | 0.99 (0.81 to 1.22) | 0.918 | 0.90 (0.67 to 1.22) | 0.510 |
| WISC 8y (total IQ score) | 3078 | 0.98 (0.97 to 0.99) | 0.003** | 0.97 (0.96 to 0.99) | 0.009** |
|  |  |  |  |  |  |
| Mental wellbeing 23y (WEMWBS composite score) | 2749 | 0.99 (0.97 to 1.02) | 0.504 | 1.02 (0.98 to 1.06) | 0.400 |
| Moderate or severe depressive episode 24y (ICD-10 diagnosis) | 3784 | 1.10 (0.58 to 2.07) | 0.775 | 1.45 (0.61 to 3.43) | 0.400 |
| Generalised anxiety disorder 24y (ICD-10 diagnosis) | 3773 | 1.19 (0.69 to 2.07) | 0.535 | 1.55 (0.72 to 3.32) | 0.259 |
| Social rapport 24y  (interviewer-rated: 1=no connection to 5=very good rapport) | 3822 | 0.15 (0.12 to 0.19) | 1.59x10^-52^*** | 0.14 (0.10 to 0.19) | 6.08x10^-31^*** |
|  |  |  |  |  |  |
| Family history of schizophrenia (1^st^ or 2^nd^ degree relatives) | 2693 | 1.10 (0.34 to 3.56) | 0.871 | 1.93 (0.45 to 8.19) | 0.375 |
| Schizophrenia PRS | 2503 | 1.25 (0.98 to 1.59) | 0.068 | 1.01 (0.69 to 1.47) | 0.971 |
| Psychotic experiences PRS (middle-older aged adults) | 2513 | 0.80 (0.63 to 1.01) | 0.062 | 0.73 (0.50 to 1.06) | 0.100 |
| Depression PRS | 2513 | 1.02 (0.81 to 1.29) | 0.865 | 1.02 (0.70 to 1.49) | 0.917 |
| Anxiety PRS | 2513 | 1.02 (0.80 to 1.30) | 0.862 | 0.96 (0.65 to 1.42) | 0.830 |
| Neuroticism PRS | 2513 | 0.98 (0.78 to 1.24) | 0.886 | 1.06 (0.73 to 1.53) | 0.774 |
| Autism PRS | 2513 | 1.08 (0.86 to 1.37) | 0.499 | 1.10 (0.76 to 1.60) | 0.603 |
|  |  |  |  |  |  |
| OR, odds ratio; 95%CI, 95% confidence interval; CSE, Certificate of Secondary Education; WISC, Wechsler Intelligence Scale for Children; WEMWBS, Warwick-Edinburgh Mental Well-being Scale; PRS, polygenic risk score.  a. Adjusted for sex (except the analysis of sex). PRS analyses using PRS standardised scores based on a GWAS discovery sample threshold of pt0.05, restricted to white ethnicity, adjusted for sex and 10 population genetic ancestry principal components.  *p<0.05, **p<0.01, ***p<0.001, two-tailed. | | | | | |

| Table S4. Logistic regression analysis of broad and narrow disorganised dimension on demographics, developmental risk factors, affective disorders, quality of life and social rapport variables^a^ | | | | | |
| --- | --- | --- | --- | --- | --- |
| Independent variable | n | Broad disorganised dimension  (score 0 vs 1-2) | | Narrow disorganised dimension  (score 0-1 vs 2) | |
|  |  | OR (95%CI) | p-value | OR (95%CI) | p-value |
| Male sex | 3824 | 1.25 (0.75 to 2.08) | 0.398 | 3.92 (1.01 to 15.17) | 0.048* |
| Non-white ethnicity | 3430 | 2.77 (0.84 to 9.09) | 0.093 | Not calculated: all 10 participants with Dis=2 of white ethnicity |  |
|  |  |  |  |  |  |
| Mother’s highest educational qualification (CSE to degree) | 3434 | 0.91 (0.73 to 1.14) | 0.417 | 0.88 (0.53 to 1.48) | 0.636 |
| Social class at birth based on mother’s occupation (I [Professional] to V [Unskilled]) | 3062 | 0.87 (0.65 to 1.16) | 0.335 | 0.92 (0.49 to 1.76) | 0.810 |
| Social class at birth based on partner’s occupation (I [Professional] to V [Unskilled]) | 2826 | 1.26 (0.97 to 1.63) | 0.079 | 1.18 (0.65 to 2.12) | 0.589 |
|  |  |  |  |  |  |
| Maternal smoking at start of pregnancy (no. times/day) | 2762 | 1.01 (0.97 to 1.05) | 0.669 | 1.02 (0.94 to 1.10) | 0.679 |
| Birthweight (grams)  [standardised] | 3501 | 0.94 (0.70 to 1.25) | 0.647 | 0.68 (0.38 to 1.20) | 0.180 |
| WISC 8y (total IQ score) | 3080 | 0.97 (0.96 to 0.99) | 0.003** | 0.95 (0.91 to 0.98) | 0.003** |
|  |  |  |  |  |  |
| Mental wellbeing 23y (WEMWBS composite score) | 2750 | 0.99 (0.96 to 1.03) | 0.662 | 0.95 (0.88 to 1.02) | 0.146 |
| Moderate or severe depressive episode 24y (ICD-10 diagnosis) | 3786 | 2.97 (1.52 to 5.82) | 0.001** | 1.75 (0.22 to 14.06) | 0.600 |
| Generalised anxiety disorder 24y (ICD-10 diagnosis) | 3775 | 1.97 (0.99 to 3.93) | 0.055 | 2.91 (0.61 to 13.95) | 0.182 |
| Social rapport 24y  (interviewer-rated: 1=no connection to 5=very good rapport) | 3823 | 0.10 (0.07 to 0.14) | 2.13x10^-35^*** | 0.16 (0.09 to 0.31) | 2.84x10^-8^*** |
|  |  |  |  |  |  |
| Family history of schizophrenia (1^st^ or 2^nd^ degree relatives) | 2693 | 1.56 (0.37 to 6.59) | 0.544 | 3.62 (0.45 to 29.39) | 0.228 |
| Schizophrenia PRS | 2503 | 0.98 (0.70 to 1.35) | 0.880 | 1.17 (0.59 to 2.31) | 0.661 |
| Psychotic experiences PRS (middle-older aged adults) | 2513 | 1.02 (0.74 to 1.41) | 0.895 | 1.21 (0.62 to 2.37) | 0.577 |
| Depression PRS | 2513 | 1.53 (1.09 to 2.16) | 0.014* | 1.32 (0.67 to 2.61) | 0.425 |
| Anxiety PRS | 2513 | 1.22 (0.88 to 1.69) | 0.227 | 0.99 (0.51 to 1.93) | 0.975 |
| Neuroticism PRS | 2513 | 1.01 (0.74 to 1.39) | 0.947 | 0.93 (0.49 to 1.78) | 0.833 |
| Autism PRS | 2513 | 0.94 (0.68 to 1.29) | 0.701 | 0.80 (0.42 to 1.55) | 0.512 |
|  |  |  |  |  |  |
| OR, odds ratio; 95%CI, 95% confidence interval; CSE, Certificate of Secondary Education; WISC, Wechsler Intelligence Scale for Children; WEMWBS, Warwick-Edinburgh Mental Well-being Scale; PRS, polygenic risk score.  a. Adjusted for sex (except the analysis of sex). PRS analyses using PRS standardised scores based on a GWAS discovery sample threshold of pt0.05, restricted to white ethnicity, adjusted for sex and 10 population genetic ancestry principal components.  *p<0.05, **p<0.01, ***p<0.001, two-tailed. | | | | | |

| Table S5. Linear regression analysis of CAPE self-rated negative symptoms on demographics, developmental risk factors, affective disorders, quality of life and social rapport variables^a^ | | | | | |
| --- | --- | --- | --- | --- | --- |
| Independent variable | n | CAPE negative symptoms sum score  (with square-root transformation) | |  | |
|  |  | β (95%CI) | p-value |  |  |
| Male sex | 3821 | 0.13 (0.06 to 0.20) | 0.000581*** |  |  |
| Non-white ethnicity | 3435 | 0.14 (-0.12 to 0.40) | 0.294 |  |  |
|  |  |  |  |  |  |
| Mother’s highest educational qualification (CSE to degree) | 3440 | -0.03 (-0.07 to -0.00) | 0.037* |  |  |
| Social class at birth based on mother’s occupation (I [Professional] to V [Unskilled]) | 3073 | 0.03 (-0.00 to 0.07) | 0.070 |  |  |
| Social class at birth based on partner’s occupation (I [Professional] to V [Unskilled]) | 2821 | 0.02 (-0.02 to 0.05) | 0.361 |  |  |
|  |  |  |  |  |  |
| Maternal smoking at start of pregnancy (no. times/day) | 2756 | 0.01 (0.00 to 0.01) | 0.007** |  |  |
| Birthweight (grams)  [standardised] | 3505 | 0.02 (-0.02 to 0.06) | 0.401 |  |  |
| WISC 8y (total IQ score) | 3072 | 0.00 (-0.00 to 0.00) | 0.479 |  |  |
|  |  |  |  |  |  |
| Mental wellbeing 23y (WEMWBS composite score) | 2759 | -0.07 (-0.07 to -0.06) | 3.39x10^-185^*** |  |  |
| Moderate or severe depressive episode 24y (ICD-10 diagnosis) | 3794 | 1.48 (1.35 to 1.61) | 1.73x10^-107^*** |  |  |
| Generalised anxiety disorder 24y (ICD-10 diagnosis) | 3785 | 1.17 (1.05 to 1.29) | 1.32x10^-82^*** |  |  |
| Social rapport 24y  (interviewer-rated: 1=no connection to 5=very good rapport) | 3664 | -0.14 (-0.19 to -0.10) | 1.64x10^-9^*** |  |  |
|  |  |  |  |  |  |
| Family history of schizophrenia (1^st^ or 2^nd^ degree relatives) | 2684 | 0.21 (-0.02 to 0.44) | 0.077 |  |  |
| Schizophrenia PRS | 2503 | 0.03 (-0.02 to 0.07) | 0.264 |  |  |
| Psychotic experiences PRS (middle-older aged adults) | 2515 | 0.06 (0.01 to 0.10) | 0.011* |  |  |
| Depression PRS | 2515 | 0.10 (0.06 to 0.15) | 0.000006*** |  |  |
| Anxiety PRS | 2515 | 0.09 (0.04 to 0.13) | 0.00010*** |  |  |
| Neuroticism PRS | 2515 | 0.11 (0.06 to 0.15) | 0.000003*** |  |  |
| Autism PRS | 2515 | 0.09 (0.05 to 0.13) | 0.000063*** |  |  |
|  |  |  |  |  |  |
| β, beta coefficient; 95%CI, 95% confidence interval; CAPE, Community Assessment of Psychic Experiences; CSE, Certificate of Secondary Education; WISC, Wechsler Intelligence Scale for Children; WEMWBS, Warwick-Edinburgh Mental Well-being Scale; PRS, polygenic risk score.  a. Adjusted for sex (except the analysis of sex). PRS analyses using PRS standardised scores based on a GWAS discovery sample threshold of pt0.05, restricted to white ethnicity, adjusted for sex and 10 population genetic ancestry principal components.  *p<0.05, **p<0.01, ***p<0.001, two-tailed. | | | | | |

| Table S6. Descriptive statistics for associations between psychotic experience dimensions and standardised schizophrenia polygenic risk score (PRS)^a^ | | | |
| --- | --- | --- | --- |
| Phenotype | Phenotype score, and schizophrenia PRS n, mean, sd | | |
|  |  |  |  |
| Positive dimension (0-2)  (n = 2521) | Score 0 = neither experience present  n = 2397  PRS mean (sd) = -0.07 (1.00) | Score 1 = one experience present  n = 106  PRS mean (sd) = -0.10 (0.93) | Score 2 = both experiences present  n = 18  PRS mean (sd) = 0.04 (1.14) |
| Negative dimension (0-2)  (n = 2503) | Score 0 = neither experience present  n = 2432  PRS mean (sd) = -0.08 (1.00) | Score 1 = one experience present  n = 44  PRS mean (sd) = 0.27 (0.92) | Score 2 = both experiences present  n = 27  PRS mean (sd) = -0.04 (0.86) |
| Disorganised dimension (0-2)  (n = 2503) | Score 0 = neither experience present  n = 2465  PRS mean (sd) = -0.07 (1.00) | Score 1 = one experience present  n = 29  PRS mean (sd) = -0.13 (1.00) | Score 2 = both experiences present  n = 9  PRS mean (sd) = 0.10 (0.98) |
|  |  |  |  |
| Self-rated negative dimension (0-30)  (n = 2503) | Score 0-10 = low  n = 1868  PRS mean (sd) = -0.08 (0.99) | Score 11-20 = medium  n = 550  PRS mean (sd) = -0.04 (1.02) | Score 21-30 = high  n = 85  PRS mean (sd) = -0.08 (1.04) |
| a. Restricted to participants of white ethnicity and where there was data on schizophrenia PRS and population genetic ancestry principal components. The standardised polygenic risk score (PRS) gives the number of standard deviations from the mean. | | | |

| Table S7. Logistic regression analysis of individual interviewer-rated psychotic experiences on schizophrenia polygenic risk score (PRS)^a^ | | | |
| --- | --- | --- | --- |
| Dependent variable  (experience/symptom) | n total; n with experience/symptom present | Schizophrenia PRS | |
|  |  | OR (95%CI) | p-value |
| *Positive psychotic experiences* |  |  |  |
| Hallucinations  (any present) | 2528; 95 | 1.01 (0.82 to 1.24) | 0.946 |
| Delusions (including thought interference) (any present) | 2531; 50 | 1.07 (0.81 to 1.42) | 0.650 |
|  |  |  |  |
| *Negative symptoms* |  |  |  |
| Reduced interaction/speech | 2504; 47 | 1.12 (0.84 to 1.50) | 0.449 |
| Restricted affect | 2504; 51 | 1.23 (0.93 to 1.63) | 0.144 |
|  |  |  |  |
| *Disorganised symptoms* |  |  |  |
| Incoherent speech | 2504; 20 | 0.82 (0.52 to 1.28) | 0.370 |
| Odd/inappropriate behaviour | 2503; 27 | 1.19 (0.80 to 1.77) | 0.385 |
|  |  |  |  |
| a. PRS analyses using PRS standardised scores based on a GWAS discovery sample threshold of pt0.05, restricted to white ethnicity, adjusted for sex and 10 population genetic ancestry principal components.  *p<0.05, **p<0.01, ***p<0.001, two-tailed. | | | |

| Table S8. Logistic regression analysis of psychotic disorder on demographics, developmental risk factors, affective disorders, quality of life and social rapport variables^a^ | | | | | |
| --- | --- | --- | --- | --- | --- |
| Independent variable | n | Psychotic disorder 12-24y  (absent/present) | |  | |
|  |  | OR (95%CI) | p-value |  |  |
| Male sex | 3889 | 0.62 (0.40 to 0.94) | 0.025* |  |  |
| Non-white ethnicity | 3485 | 0.46 (0.06 to 3.37) | 0.447 |  |  |
|  |  |  |  |  |  |
| Mother’s highest educational qualification (CSE to degree) | 3489 | 0.97 (0.82 to 1.16) | 0.768 |  |  |
| Social class at birth based on mother’s occupation (I [Professional] to V [Unskilled]) | 3110 | 1.10 (0.91 to 1.32) | 0.334 |  |  |
| Social class at birth based on partner’s occupation (I [Professional] to V [Unskilled]) | 2870 | 1.05 (0.86 to 1.28) | 0.642 |  |  |
|  |  |  |  |  |  |
| Maternal smoking at start of pregnancy (no. times/day) | 2805 | 1.04 (1.01 to 1.07) | 0.003** |  |  |
| Birthweight (grams)  [standardised] | 3558 | 1.15 (0.91 to 1.46) | 0.228 |  |  |
| WISC 8y (total IQ score) | 3126 | 0.98 (0.97 to 0.99) | 0.004** |  |  |
|  |  |  |  |  |  |
| Mental wellbeing 23y (WEMWBS composite score) | 2794 | 0.91 (0.88 to 0.93) | 1.26x10^-15^*** |  |  |
| Moderate or severe depressive episode 24y (ICD-10 diagnosis) | 3846 | 6.76 (4.44 to 10.29) | 5.67x10^-19^*** |  |  |
| Generalised anxiety disorder 24y (ICD-10 diagnosis) | 3836 | 6.13 (4.07 to 9.22) | 3.49x10^-18^*** |  |  |
| Social rapport 24y  (interviewer-rated: 1=no connection to 5=very good rapport) | 3825 | 0.67 (0.53 to 0.84) | 0.0004*** |  |  |
|  |  |  |  |  |  |
| Family history of schizophrenia (1^st^ or 2^nd^ degree relatives) | 2735 | 0.45 (0.06 to 3.28) | 0.431 |  |  |
| Schizophrenia PRS | 2541 | 1.10 (0.85 to 1.42) | 0.483 |  |  |
| Psychotic experiences PRS (middle-older aged adults) | 2551 | 1.21 (0.94 to 1.56) | 0.147 |  |  |
| Depression PRS | 2551 | 1.30 (1.00 to 1.67) | 0.047* |  |  |
| Anxiety PRS | 2551 | 1.22 (0.95 to 1.57) | 0.124 |  |  |
| Neuroticism PRS | 2551 | 1.32 (1.02 to 1.70) | 0.034* |  |  |
| Autism PRS | 2551 | 0.91 (0.71 to 1.16) | 0.444 |  |  |
|  |  |  |  |  |  |
| OR, odds ratio; 95%CI, 95% confidence interval; CSE, Certificate of Secondary Education; WISC, Wechsler Intelligence Scale for Children; WEMWBS, Warwick-Edinburgh Mental Well-being Scale; PRS, polygenic risk score.  a. Adjusted for sex (except the analysis of sex). PRS analyses using PRS standardised scores based on a GWAS discovery sample threshold of pt0.05, restricted to white ethnicity, adjusted for sex and 10 population genetic ancestry principal components.  *p<0.05, **p<0.01, ***p<0.001, two-tailed. | | | | | |

| Table S9. Results of exploratory factor analysis of individual positive psychotic experiences^a^ | | |
| --- | --- | --- |
| Positive psychotic experiences | Factor loadings^b^ | |
|  | Factor 1  (First rank delusions) | Factor 2  (Paranoid) |
| Auditory hallucinations | .092 | **.580** |
| Visual hallucinations | -.016 | **.530** |
| Delusions of being spied on | .087 | **.674** |
| Delusions of persecution | .144 | **.642** |
| Delusions of thoughts being read | .305 | .408 |
| Delusions of reference | .377 | .489 |
| Delusions of control | **.752** | .158 |
| Grandiose delusions | .262 | .486 |
| Other delusions | -.134 | .264 |
| Thought broadcast | **.631** | -.053 |
| Thought insertion | **.680** | .206 |
| Thought withdrawal | **.702** | .048 |
| a. Positive psychotic experiences assessed by interviewer at 24y for timeframe of 12-24y and rated as 0=absent, 1=uncertain (including if occurred only while waking up/going to sleep, with fever, or under the influence of alcohol/drugs), or 2=present. Psychotic experiences in 3862 participants analysed using principal components analysis with varimax rotation - loadings for two resulting factors shown.  b. Factor loadings that were high (>0.5) and distinct (without marked cross-loadings) shown in bold. | | |
